# Brain Endothelial Erythrophagocytosis and Hemoglobin Transmigration Across Brain Endothelium: Implications for Pathogenesis of Cerebral Microbleeds

**DOI:** 10.3389/fncel.2018.00279

**Published:** 2018-09-06

**Authors:** Rudy Chang, Juan Castillo, Alexander C. Zambon, Tatiana B. Krasieva, Mark J. Fisher, Rachita K. Sumbria

**Affiliations:** ^1^Department of Biopharmaceutical Sciences, School of Pharmacy and Health Sciences, Keck Graduate Institute, Claremont, CA, United States; ^2^Department of Neuroscience, Claremont McKenna College, Claremont, CA, United States; ^3^Beckman Laser Institute, University of California, Irvine, Irvine, CA, United States; ^4^Departments of Neurology and Pathology & Laboratory Medicine, University of California, Irvine, Irvine, CA, United States

**Keywords:** erythrophagocytosis, cerebral microbleeds, brain endothelial cells, hemoglobin, red blood cells, transmigration

## Abstract

Peripheral endothelial cells are capable of erythrophagocytosis, but data on brain endothelial erythrophagocytosis are limited. We studied the relationship between brain endothelial erythrophagocytosis and cerebral microhemorrhage, the pathological substrate of MRI-demonstrable cerebral microbleeds. To demonstrate the erythrophagocytic capability of the brain endothelium, we studied the interactions between brain endothelial cells and red blood cells exposed to oxidative stress *in vitro*, and developed a new *in vitro* cerebral microbleeds model to study the subsequent passage of hemoglobin across the brain endothelial monolayer. Using multiple approaches, our results show marked brain endothelial erythrophagocytosis of red blood cells exposed to oxidative stress compared with control red blood cells *in vitro*. This brain endothelial erythrophagocytosis was accompanied by passage of hemoglobin across the brain endothelial monolayer with unaltered monolayer integrity. *In vivo* and confocal fluorescence microscopy studies confirmed the extravasation of RBC exposed to oxidative stress across brain endothelium. These findings, demonstrating erythrophagocytosis mediated by the brain endothelial monolayer and the subsequent passage of iron-rich hemoglobin *in vitro* and RBC *in vivo*, may have implications for elucidating mechanisms involved in the development of cerebral microbleeds that are not dependent on disruption of the microvasculature.

## Introduction

Cerebral microhemorrhages are tiny deposits of blood degradation products in the brain and are the pathological substrate of cerebral microbleeds (CMB). CMB are associated with increasing age, cerebrovascular diseases, hypertension, and chronic kidney disease (Lau et al., 2017). Mechanistically, it is widely accepted that loss of vascular integrity due to an underlying disruption of the cerebral microvasculature causes CMB development, and these are classified as primary microbleeds (Fisher, 2014). Other mechanisms involved in CMB development include hemorrhagic infarction/microinfarction, and these are classified as secondary microbleeds (Fisher, 2013, 2014). Besides the involvement of hemorrhagic and ischemic processes in the development of CMB, there has been some indication that release of iron from oligodendrocytes during ischemia may result in MRI-demonstrable CMB (Janaway et al., 2014). Further, the existence of *pseudo-microbleeds*, a phenomenon in which iron-rich hemosiderin from red blood cells (RBC) produces signatures of CMB in the absence of vascular disruption, has been proposed (Fisher, 2014).

A recent study showed that brain endothelium have phagocytic properties and that brain vascular endothelial cells are capable of engulfing and translocating intracranial emboli into the brain extravascular space, a process termed angiophagy (Grutzendler et al., 2014). The concept of angiophagy has some similarities to endothelial erythrophagocytosis, a recently described process by which injured or aged erythrocytes are ingested by endothelial cells, undergo hemolysis, and are cleared from the blood circulation (Fens et al., 2010). Endothelial erythrophagocytosis has recently been reported by peripheral endothelial cells using human umbilical vein endothelial (HUVEC) and hepatic sinusoidal endothelial cells *in vitro*, and has further been confirmed *in vivo* (Fens et al., 2010, 2012; Lee et al., 2011).

In the cerebral milieu, macrophage-mediated erythrophagocytosis has been well-documented (Zhao et al., 2009), and the involvement of macrophages in CMB is known (Fisher et al., 2010). Besides macrophages, pericytes are known to have phagocytic function and there is some evidence of pericyte mediated erythrophagocytosis in the brain (Fisher et al., 2010). Phagocytosis of parasitized RBC by brain endothelium within an inflammatory milieu has been reported in *in vitro* studies of cerebral malaria (Jambou et al., 2010). Involvement of brain endothelium in phagocytosis of non-parasitized aged and/or injured RBC and subsequent transmigration of RBC or RBC degradation products (hemoglobin) into the brain has not been reported. It has been proposed that iron-rich extravasated RBC or RBC degradation products may produce MRI signatures of CMB in the absence of vascular disruption (pseudo-microbleeds) (Fisher, 2014).

The above prompted us to investigate the role of the brain endothelium in mediating erythrophagocytosis of aged and/or injured RBC, and subsequent passage of RBC and/or RBC degradation product hemoglobin into the brain. In the current proof-of-concept study to demonstrate brain endothelial erythrophagocytosis, RBC were treated with phosphate buffer saline (PBS) or tert-butylhydroperoxide (t-BHP), an oxidative stressor that results in the exposure of RBC phosphatidylserine (PS) to mimic aged and/or injured RBC marked for phagocytic removal from the circulation (Schroit et al., 1985). Using multiple approaches, our results show robust adhesion and engulfment (erythrophagocytosis) of t-BHP-RBC by brain endothelial cells (bEND3) compared with PBS-RBC. We developed a new CMB *in vitro* model system to study passage of RBC degradation product hemoglobin across bEND3 cells, and show passage of hemoglobin from t-BHP-treated RBC across the bEND3 monolayer with unaltered monolayer integrity. Our *in vitro* results were corroborated by our *in vivo* findings of extrusion of fluorescently-labeled RBC across the mouse brain endothelium. These results may have important implications for elucidating mechanisms involved in the development of CMB demonstrated on brain MRI.

## Materials and methods

### Red blood cell preparation and treatment

Purified RBC in Alsevers solution were derived from 2 to 3 month-old male BALB/c mice (BioReclamation IVT, New York, NY). RBC were washed and resuspended in sterile PBS (without Ca^2+^and Mg^2+^) before experimental treatment with PBS (control; incubation time: 30 min), lipopolysaccharide derived from gram negative bacterium Salmonella Typhimurium (LPS, 10 μg/mL; incubation time: 1 h; an inflammatory stimulus), neuraminidase enzyme from vibrio cholerae (0.125 U or 0.5 U; incubation time: 1 h; for senescence induction) or various concentrations (0.3, 1.0, 3.0 mM) of t-BHP (incubation time: 30 min; an oxidative stressor) at 37°C. LPS, neuraminidase, and t-BHP were selected for the current study based on previous studies using peripheral endothelial cells (Tissot Van Patot et al., 1996; Fens et al., 2012; Yang et al., 2014). After treatment, RBC were washed and resuspended in sterile PBS to desired concentrations described below. All the above materials were purchased from Sigma-Aldrich, St. Louis, MO.

### Brain endothelial cell culture

Murine brain microvascular endothelial cells (bEND3 cells; American Type Culture Collection, Manassas, VA) between passages 22 and 31 were grown on T75 flasks in Delbucco's Modified Eagle's Medium (DMEM) containing 0.45% glucose, 0.37% NaHCO_3_, 4 mM Glutamine, 10% fetal bovine serum, and 100 μg/ml penicillin/streptomycin (Sigma, St. Louis, MO). After reaching 70–80% confluency, cells were subcultured in complete DMEM and seeded onto 6-well plates (5 × 10^5^ bEND3 cells per well), 24 well plates with 0.2% gelatin coated glass cover slips (1 × 10^5^ bEND3 cells per coverslip), or 12-well transwells with 3 μm pore polyester membrane inserts with a surface area of 1.12 cm^2^ (Corning, New York, NY) (1 × 10^5^ bEND3 cells per well). For all the experiments, 5 × 10^6^ treated RBC were incubated with the seeded bEND3 cells for up to 24 h at 37°C in 5% CO_2_. For initial experiments, 24 h after initial seeding of bEND3 cells grown on 24 well plates with 0.2% gelatin coated cover glass, bEND3 cells were primed with or without 100 ng/mL of LPS for another 24 h before being incubated with treated RBC to determine the effect of endothelial activation on RBC-bEND3 interactions.

### Annexin V-FITC labeling

To quantify the percentage of treated RBC with PS exposure, t-BHP- and PBS-treated RBC were labeled with annexin-V-FITC (BioLegend, San Diego, CA) as per the manufacturer's instructions. Briefly, after wash and resuspension of RBC in sterile PBS, 1 × 10^6^ RBC were incubated in FITC-labeled annexin-V and 1.2 mM Ca^2+^ for 30 min in the dark. After washing and resuspending in sterile PBS, RBC were quantified with a flow cytometer (Accuri C6 Plus, BD, Franklin lakes, NJ) using logarithmic gain for light scatter and fluorescence channels with gating and background settings defined through the PBS/control RBC.

### Hematoxylin and eosin staining

Twenty-four hours after initial bEND3 seeding on 0.2% gelatin coated glass coverslips in 24 well plates, the cells were incubated with RBC treated either with PBS or t-BHP for 3 h, 18 h and 24 h. Glass cover-slips were then fixed with 4% paraformaldehyde (PFA) for 15 min on ice followed by hematoxylin and eosin (H&E) staining. Briefly, each coverslip was washed with ice cold PBS, stained with hematoxylin for 10 min followed by Scott's Tap Water wash, stained with eosin Y for 8 min, and washed with ethanol and dipped in xylene before mounting. Six fields of view per coverslip were manually quantified by an observer blinded to the treatment using light microscopy at a 20X magnification to estimate: RBC adhesion: RBC to bEND3 ratio (expressed as %) and engulfment: % of bEND3 that were positive for RBC engulfment.

### Diaminofluorene assay

H&E staining results were confirmed using a colorimetric assay which is based on the pseudoperoxidase activity of hemoglobin using 2,7-diaminofluorene (DAF) (Sigma, St. Louis, MO) substrate and hydrogen peroxide (Gebran et al., 1992). For this, bEND3 cells seeded onto 6-well plates at 5 × 10^5^ cells per well for 72 h at 37°C in 5% CO_2_ were used. After 72 h, cell media was changed from the FBS-supplemented DMEM to strictly FBS-free DMEM. After media change, RBC treated with PBS or t-BHP were incubated with the bEND3 cells for 24 h. bEND3 cells without RBC served as control (bEND3 only group). After incubation, the supernatant media of the wells was aspirated, wells were washed with distilled water to lyse attached RBC (RBC that are not engulfed by endothelial cells) and incubated with 0.2M Tris-HCl buffer containing 6M urea lysis solution for 30 min, followed by circular scrubbing with cell scrappers to further lyse bEND3 cells to release hemoglobin. DAF dye stock solution (10 mg/mL in 90% acetic acid) was added to 10 mL of 6M urea with 100 μL of hydrogen peroxide to make a DAF working solution which was used to react with 100 μL of supernatant of each well prepared above and immediately read on a SpectraMax 384 microplate reader (Molecular Devices, Sunnyvale, CA) at 620 nm. A hemoglobin standard (Lee Biosolutions, Maryland Heights, MO) was used to estimate the amount of hemoglobin which is an indicator of the extent of erythrophagocytosis. The amount of hemoglobin was expressed as % of hemoglobin detected with the bEND3 only group. Published studies have shown that high concentrations of t-BHP convert hemoglobin to methemoglobin (Murakami and Mawatari, 2003), which is not detected by the DAF assay. This was further confirmed in our lab (data not shown) and as a result, the DAF assay was performed using the lowest dose of t-BHP (0.3 mM).

### Red blood cell incubation viability

The viability of PBS- and t-BHP-treated RBC in cell culture incubation environment was assessed by incubating treated RBC (5 × 10^6^ cells per well) in DMEM or FBS-free DMEM into 24 well plates. After 24 h, 10 μL cells were collected and mixed with trypan blue at 1:1, and manually counted with a hemocytometer for both live and dead RBC based on trypan blue exclusion. Viable RBC were expressed as % of total RBC.

### Confocal microscopy

Brain endothelial cell tight-junctions were imaged and analyzed through Zonula Occludens-1 (ZO-1) staining and confocal microscopy. Twenty-four hours after initial bEND3 seeding on 0.2% gelatin coated glass coverslips in 24 well plates, the cells were incubated with RBC treated either with PBS or t-BHP for 24 h. Glass cover-slips were then fixed with 2% PFA, blocked in 5% bovine serum albumin (BSA) in PBS with 0.1% tritonX-100, incubated with ZO-1 antibody (1:100, ZO-1 Alexa Flour 488, SantaCruz Biotechnology, Dallas, TX; Antibody Registry Accession No. AB_628459) in PBS with 0.75% BSA overnight at 4°C, followed by endothelial nuclear staining with DRAQ5 (BioLegend, San Diego, CA). Cover-slips were mounted with VectaShield Hardset Mounting Medium (Vector Labs, Burlingame, CA) and imaged with a Leica SP5 confocal microscope (Leica, Wetzlar, Germany).

### Live imaging

To visualize the time-lapse of events that occurs between bEND3 cells and RBC, PBS- and t-BHP-treated RBC that were incubated with bEND3 cells on 6-well plates were placed in an incubator (37°C in 5% CO_2_) with LumaScope 720 imaging microscope (EtaLuma, Carlsbad, CA) that captured images of each well every 2.5 min over a course of 24 h. The timestamped images were then compiled into an mpeg file that illustrated the time-lapse at 3 frames per second.

### Transmigration of hemoglobin across the bEND3 monolayer

To determine the appropriate time to initiate the transmigration experiments, the integrity of the bEND3 monolayer in the transwells was assessed by performing transendothelial electrical resistance (TEER) measurements using the EVOM2 Epithelial Volt/Ohm Meter and an STX-2 electrode system (World Precision Instruments LLC, Sarasota, FL) at various time points (3, 24, 48, and 72 h) in each well.

The experimental setup of the transmigration experiment is shown in **Figure 4A**. To assess whether hemoglobin from treated RBC migrates through the endothelial monolayer, bEND3 cells were cultured onto the apical wells of the transwells with complete DMEM for 72 h (time at which maximum TEER was obtained; data not shown), followed by replacement of the complete DMEM with FBS-free DMEM. PBS- and t-BHP-treated RBC were then added to the apical wells, incubated undisturbed for 24 h at 37°C in 5% CO_2_. Endothelial cells grown on transwell inserts without RBC served as controls (bEND3 only group). After incubation, media in the basolateral well was gently mixed and 200 μL of the media was collected and lysed with 200 μL of 0.2 M Tris-HCl buffer containing 6 M urea for 15 min at RT. The basolateral aliquot was then examined for hemoglobin passage across the bEND3 monolayer using the DAF and hemoglobin standard assay described above. Passage of hemoglobin across bEND3 monolayer-free transwell insert was used to assess spontaneous migration of hemoglobin across the transwell insert. Hemoglobin amount was expressed as % bEND3 only group (control).

TEER measurements were performed in triplicates per insert before addition of RBC to the apical well (0 h) and 24 h after RBC incubation to assess monolayer integrity during the transmigration experiment. All TEER values were corrected for the blank TEER of monolayer-free transwell inserts and represented at % PBS RBC values.

### *In vivo* passage of RBC across the brain endothelium

All animal procedures were approved by UCI Institutional Animal Care and Use Committee and were carried out in compliance with University Laboratory Animal Resources regulations. Passage of t-BHP-treated RBC across the brain endothelium was visualized using postmortem confocal fluorescence microscopy. Adult male Tie2-GFP mice (10–12 week old, Jackson Laboratory, Bar Harbor, ME) with green fluorescent protein (GFP) labeled endothelial cells were used for imaging. Briefly, autologous blood (200–250 μL) was collected from Tie2-GFP mice (*n* = 2) and RBC were purified using Ficoll-Paque (GE Healthcare, Uppsala, Sweden) gradient. After several washes in PBS, RBC were treated with PBS or 3 mM t-BHP for 30 min at 37°C as described above and stained with PKH-26 Red Fluorescent Cell Linker Kit (Sigma, St. Louis, MO) according to manufacturer's instructions and re-injected into the mice intravenously. Mice were euthanized 24 h after RBC injection using a lethal dose of Euthasol (150 mg/kg, i.p.) and whole brains were harvested for postmortem confocal fluorescence microscopy to visualize passage of RBC across the brain endothelium.

Fluorescence images of whole brains were obtained using Zeiss LSM 510 microscopy system equipped with a longworking distance Zeiss 40x, 0.8 NA water immersion objective. Laser scanning did not induce any visible damage to the tissue or noticeable bleaching of the sample. Stacks of images were acquired with the z-step (distance between consecutive imaging planes) of 2.5 μm. The maximum depth for imaging was up to 80 μm from the brain surface. The probed 3D volume was reconstructed by Zeiss LSM original software.

### Measurement of free hemoglobin from RBC

bEND3 cells (1 × 10^5^) seeded onto 24 well plates were allowed to grow to confluence and incubated with PBS- or t-BHP-treated RBC in FBS free media for 3 h, 18 h, and 24 h. At the predetermined time intervals, media was collected from each well, and placed in Amicon-100K centrifuge tubes (Fisher Scientific, Hampton, NH). After spinning for 10 min at 14,000 RPM, the filtrate at the bottom containing the free hemoglobin was collected (RBC are retained on top) and processed using the DAF assay described above to determine free hemoglobin present in the media represented as % of hemoglobin from PBS RBC (% control).

### Statistical analysis

All statistical analysis was performed using GraphPad Prism 5 (GraphPad Software Inc., La Jolla, CA). Data are presented as mean ± SEM of at least 3 independent experiments done in duplicates based on power analysis which indicated that at least 3 experiments will be needed to detect a difference of 20% between groups with a power of 80% and significance level of 5%. To compare more than 2 independent groups, one-way ANOVA with Bonferroni's correction was used. For data with more than 2 independent groups and 2 independent variables, two-way ANOVA, with or without repeated measures, was used. A two-tailed *p* < 0.05 for the entire family of comparison was considered statistically significant.

## Results

**Figure 1:**
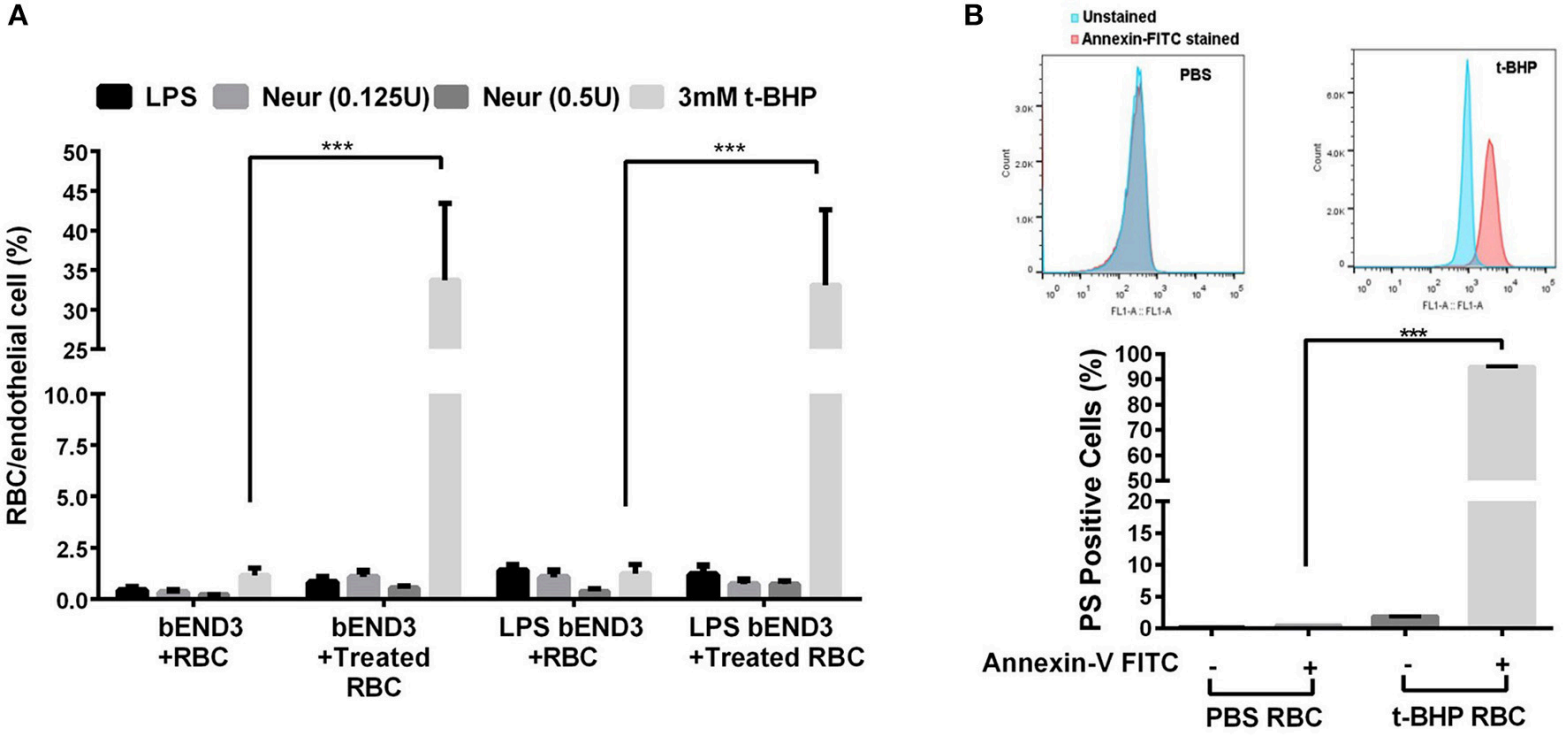
RBC adhesion to brain endothelial (bEND3 cells) under different conditions **(A)** and PS exposure in t-BHP-treated RBC **(B)**. RBC treated with PBS (control), LPS (10 μg/mL) or neuraminidase (0.125 U or 0.5 U) did not significantly alter RBC adhesion to bEND3 cells following 3 h incubation, however, 3 mM t-BHP treatment of RBC resulted in a significant increase in RBC adhesion to bEND3 cells compared with respective PBS-treated RBC. LPS treatment of bEND3 cells did not further enhance RBC adhesion under any treatment conditions. >90% of 0.3 mM t-BHP-treated RBC were PS positive compared with PBS-treated RBC as determined by flow cytometry **(B)**. Data is presented as mean ± SEM of at least 3 independent experiments. ***p < 0.001.

Three-hour incubation of PBS-treated bEND3 cells with PBS-treated RBC resulted in negligible RBC adhesion to bEND3 cells. There was a significant (*p* < 0.001) increase in the adhesion of 3 mM t-BHP-treated RBC to PBS-treated bEND3 cells compared with PBS-treated RBC incubated with PBS-treated bEND3 cells (Figure 1A). Treatment of RBC with LPS or neuraminidase (0.125U or 0.5U) did not significantly alter RBC adhesion to PBS-treated bEND3 cells compared with PBS-treated RBC incubated with PBS-treated bEND3 cells (Figure 1A). Treatment of the bEND3 cells with LPS did not have a significant effect on RBC adhesion under any condition tested in the current study (Figure 1A) or with an increase in incubation time (data not shown).

### Phosphatidylserine exposure

Studies with annexin-V FITC showed that greater than 90% of t-BHP-treated RBC are annexin-V FITC positive confirming PS exposure on the surface of t-BHP-treated RBC compared with PBS-treated RBC (Figure 1B).

### Concentration and time dependent RBC-bEND3 erythrophagocytosis (adhesion and engulfment)

**Figure 2:**
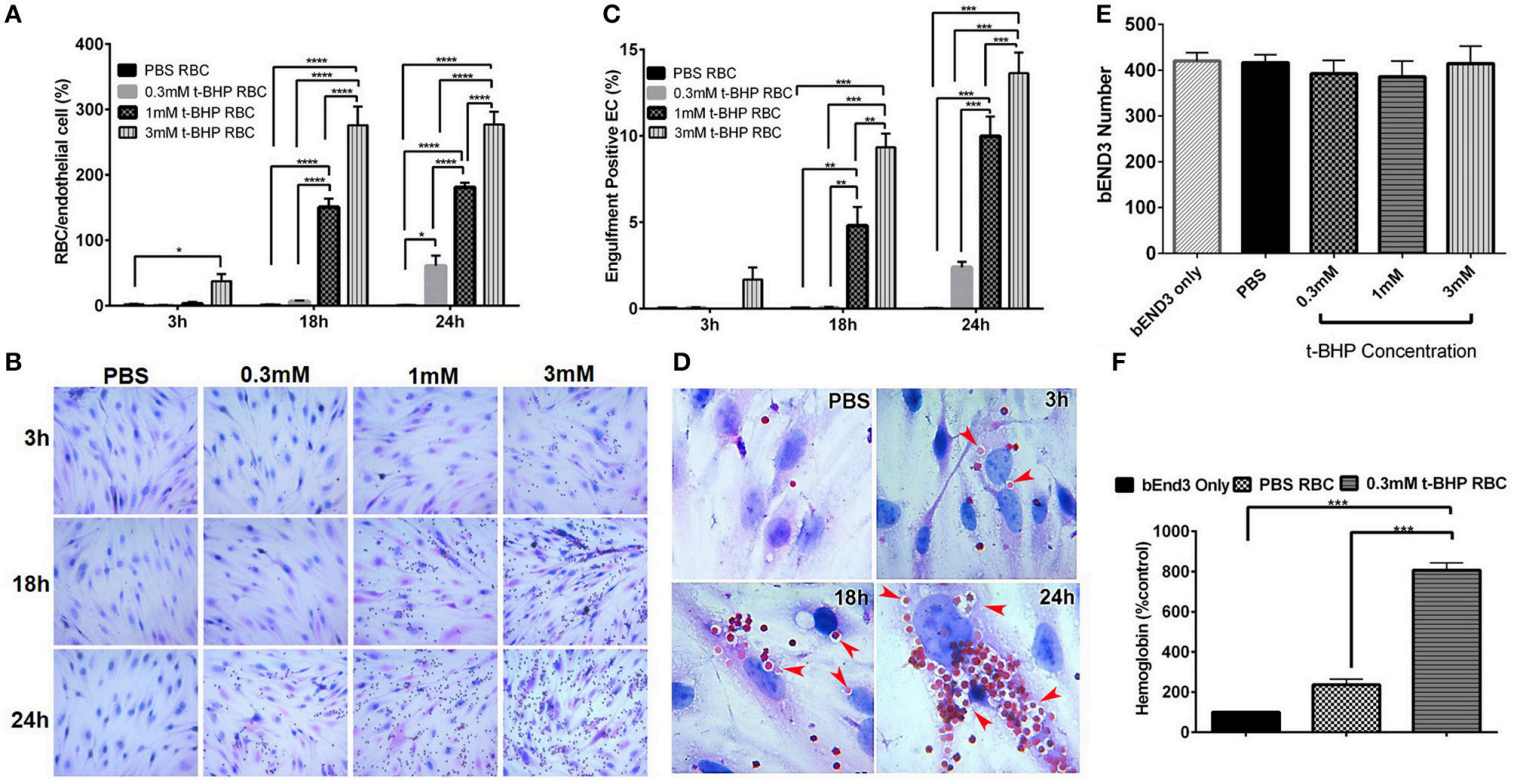
Erythrophagocytosis by brain endothelial cells. Significant time- and concentration-dependent increase in adhesion **(A,B)** and engulfment **(C)** of t-BHP-treated RBC compared with PBS-treated RBC using light microscopy. Marked time dependent increase in engulfment of t-BHP-treated RBC by bEND3 cells indicated by red arrows **(D)**. Brain endothelial erythrophagocytosis at 24 h was not associated with bEND3 degradation or loss **(E)**. Significant increase in the amount of hemoglobin retained by the bEND3 cells incubated with t-BHP-treated RBC compared with PBS-treated RBC following 24 h incubation confirming erythrophagocytosis using the DAF colorimetric method **(F)**. Data is presented as mean ± SEM of at least 3 independent experiments. *p < 0.05, **p < 0.01, ***p < 0.001, ****p < 0.0001. EC, endothelial cells.

RBC treatment with low and medium concentrations of t-BHP (0.3 and 1 mM) resulted in negligible RBC adhesion to the bEND3 cells following 3 h incubation. RBC treatment with high concentration of t-BHP on the other hand resulted in significant RBC adhesion to the bEND3 cells as early as following 3 h incubation. Overall, adhesion of t-BHP-treated RBC to bEND3 cells, increased in a concentration and time dependent manner. Maximum RBC adhesion for the 0.3 mM t-BHP concentration was observed at 24 h and RBC adhesion plateaued at 18 h for 1 mM and 3 mM t-BHP concentrations (Figure 2A,B).

Similarly, low and medium concentrations of t-BHP resulted in no engulfment of RBC by bEND3 cells following 3 h incubation. There was a trend toward an increase in the engulfment of 3 mM t-BHP-treated RBC following 3 h incubation. Engulfment of t-BHP-treated RBC also increased in a concentration and time dependent manner with maximum engulfment of t-BHP-treated RBC occurring following 24 h incubation (Figure 2C). Overall, maximum adhesion and engulfment of t-BHP-treated RBC was observed with a concentration of 3 mM. As shown in Figure 2D, multiple 3 mM t-BHP-treated RBC are seen engulfed (red arrows) by a single bEND3 cell. The average number of bEND3 cells per field of view remained unchanged with adhesion and engulfment of t-BHP-RBC over the duration of the experiment (Figure 2E) for any experimental group and viability of the PBS- and t-BHP-treated RBC in cell culture media was > 90% at 24 h (data not shown).

DAF assay that relies on the pseudoperoxidase activity of hemoglobin was used to confirm erythrophagocytosis (adhesion and engulfment) observed by microscopy. There was no significant difference in the amount of hemoglobin detected in the bEND3 cells incubated with PBS-treated RBC compared with control (bEND3 monolayer only). However, bEND3 cells incubated with t-BHP-treated RBC had a significantly higher amount of hemoglobin, an indicator of erythrophagocytosis, compared with the control and PBS-treated RBC, confirming the light microscopy results (Figure 2F).

**Figure 3:**
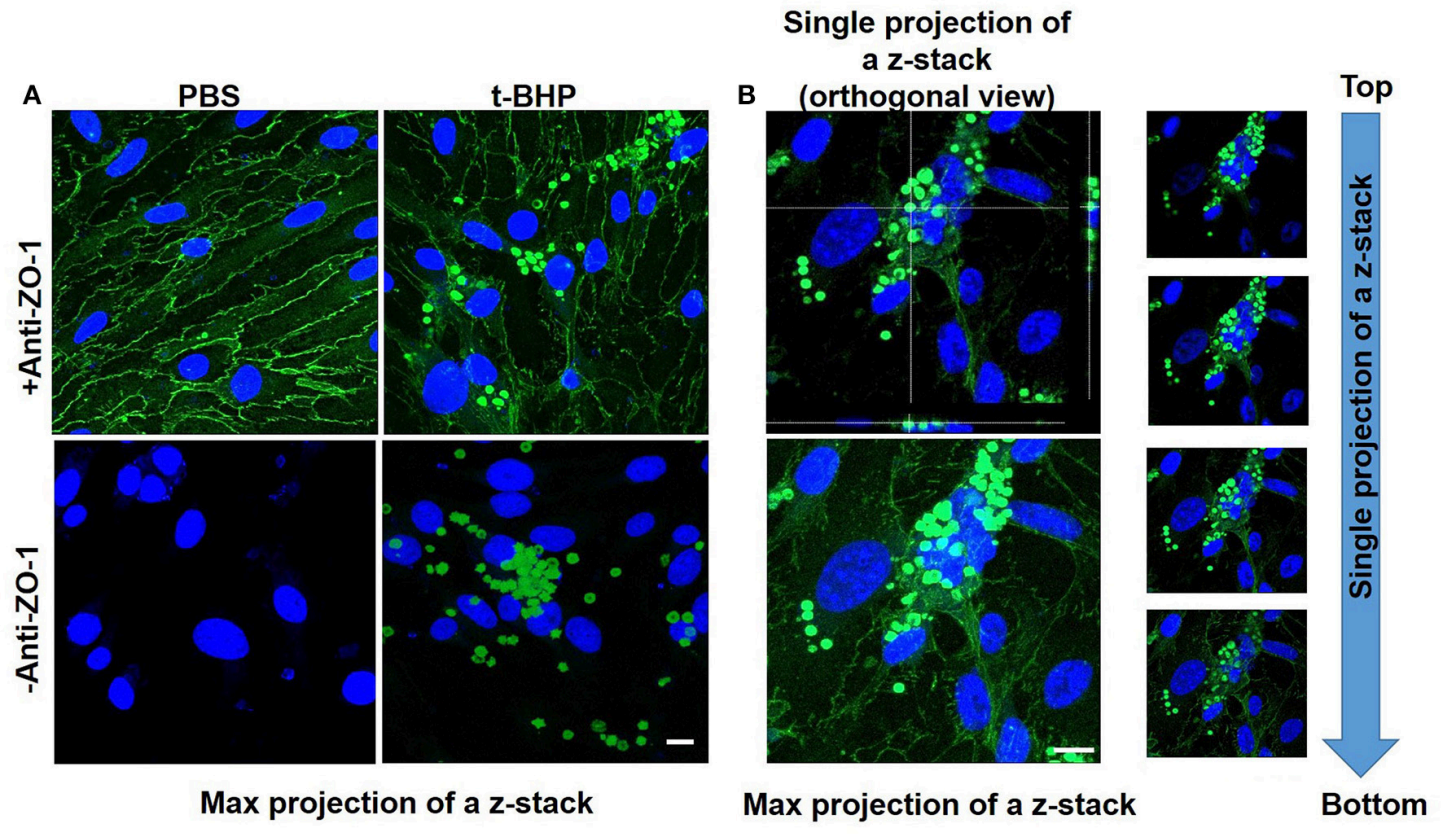
Confocal microscopy of bEND3 cells and RBC to confirm erythrophagocytosis. Maximum projection of z-stack shows adhesion of t-BHP-treated RBC to bEND3 cells and no adhesion of PBS-treated RBC (Blue = DRAQ5 stain for the endothelial nucleus, green = tight junction ZO-1 protein and autofluorescence of RBC) **(A)**. Orthogonal view of a single projection, maximum projection of a z-stack and individual images of a z-stack moving from top to bottom of a cover glass confirm the internalization of t-BHP-treated RBC by bEND3 cells **(B)**. Scale bar = 10 μm.

Adhesion and engulfment of t-BHP-treated RBC by the bEND3 monolayer was further confirmed using live cell time-lapse imaging (see Supplementary Video 1), immunofluorescence and confocal microscopy. Figure 3A shows a maximum projection of a 6 μm depth z-stack image with a step size of 0.3 μm, and confirms the presence of tight junction protein ZO-1 in the confluent bEND3 monolayer (ZO-1 tight junction protein staining shown in green and bEND3 nuclei stained with DRAQ5 shown in blue). Both PBS- and t-BHP- RBC autofluoresce (green) as shown in the figure. Adhesion and engulfment of t-BHP-treated RBC is further confirmed by orthogonal and single z-stack projections (Figure 3B).

### Transmigration of hemoglobin across the bEND3 monolayer

**Figure 4:**
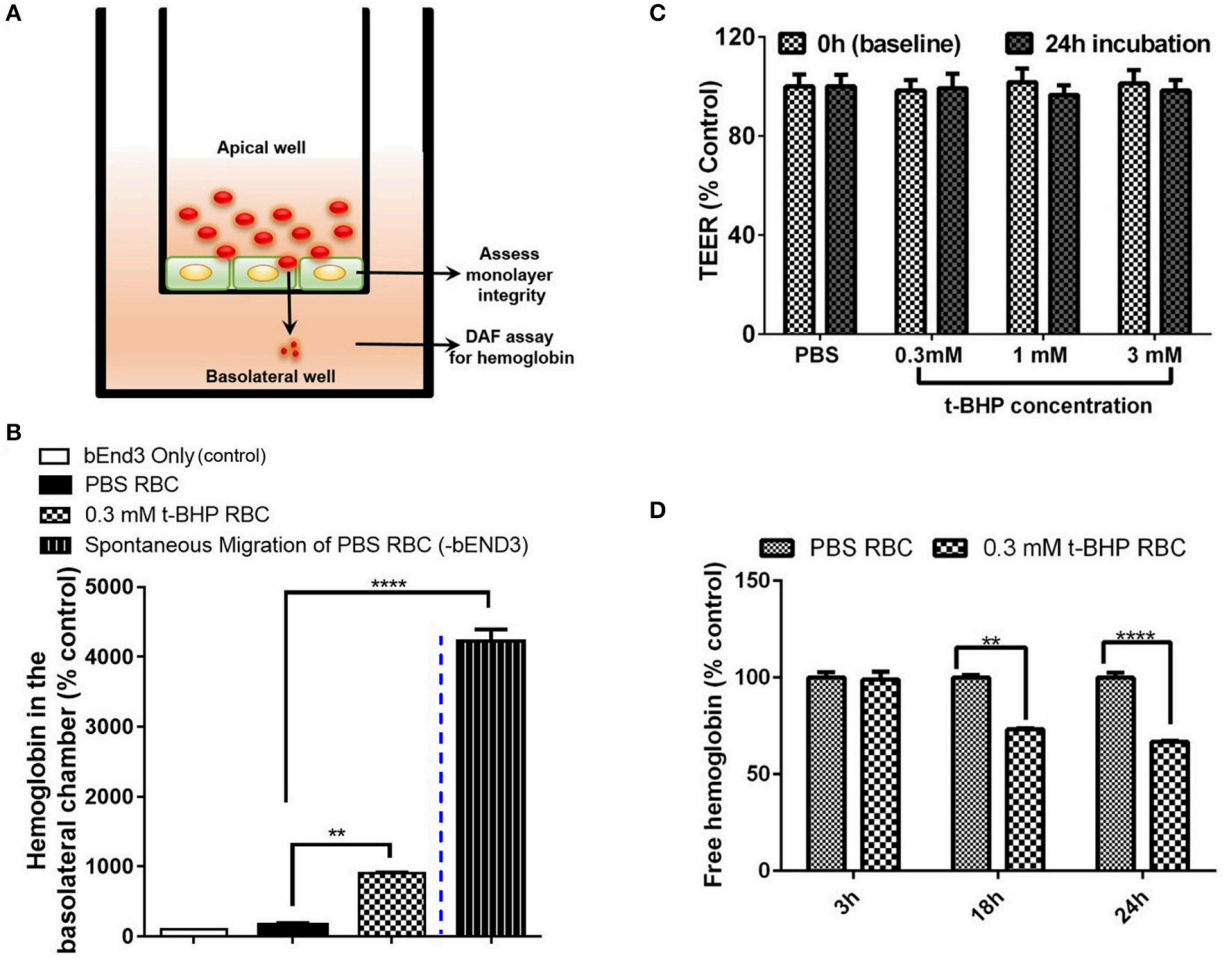
Transmigration of hemoglobin across the bEND3 monolayer. Experimental set up to detect passage of hemoglobin across the bEND3 monolayer **(A)**. Significant increase in the passage of hemoglobin across the bEND3 monolayer incubated with t-BHP-treated RBC compared with PBS-RBC. Spontaneous migration reflects passage of hemoglobin from PBS-RBC across the 3 μm pore size transwell in the absence of a bEND3 monolayer **(B)**. Passage of hemoglobin across the bEND3 monolayer grown on transwells incubated with t-BHP-RBC was not accompanied by a change in TEER; TEER measurements are presented as % of PBS-RBC values **(C)**. Free hemoglobin released by t-BHP-treated RBC is significantly lower compared with PBS-treated RBC. The bEND3 monolayer was grown on standard cell culture wells **(D)**. Data is presented as mean ± SEM of at least 3 independent experiments. **p < 0.01, ****p < 0.0001.

We developed a method to determine the passage of hemoglobin across the bEND3 monolayer using an *in vitro* transwell system. Experimental set-up for the transmigration experiment is shown in Figure 4A. Migration of hemoglobin across the bEND3 monolayer was determined using the DAF assay as described above. Spontaneous migration reflects the passage of hemoglobin from PBS-RBC across the 3 μm pore size transwell in the absence of a bEND3 monolayer. As expected, hemoglobin passage was observed in the absence of the bEND3 monolayer. However, in the presence of the bEND3 monolayer, the passage of hemoglobin from PBS-RBC across the bEND3 monolayer was the same as control (bEND3 only) indicating no passage of hemoglobin from PBS-RBC across the monolayer. There was a significant increase in the hemoglobin in the basolateral chamber of wells incubated with t-BHP-treated RBC compared with PBS-treated RBC confirming passage of hemoglobin from t-BHP-treated RBC across the bEND3 monolayer (Figure 4B).

To determine if passage of hemoglobin from the t-BHP-treated RBC was associated with changes in bEND3 monolayer integrity, we measured TEER values, before and 24 h after RBC incubation. No changes in TEER were observed for all concentrations of t-BHP compared with PBS-RBC used as control, confirming that the passage of hemoglobin from t-BHP-RBC was not associated with altered bEND3 monolayer integrity (Figure 4C). Further, to rule out the possibility of an increase in hemoglobin release with t-BHP in the apical chamber that may result in higher hemoglobin in the basolateral chamber, we determined the amount of free hemoglobin released by PBS- and t-BHP-treated RBC over the course of 24 h. We found that treating RBC with t-BHP did not increase the free hemoglobin released in the media compared with PBS-treated RBC (Figure 4D).

### Passage of RBC across the brain endothelium *in vivo*

**Figure 5:**
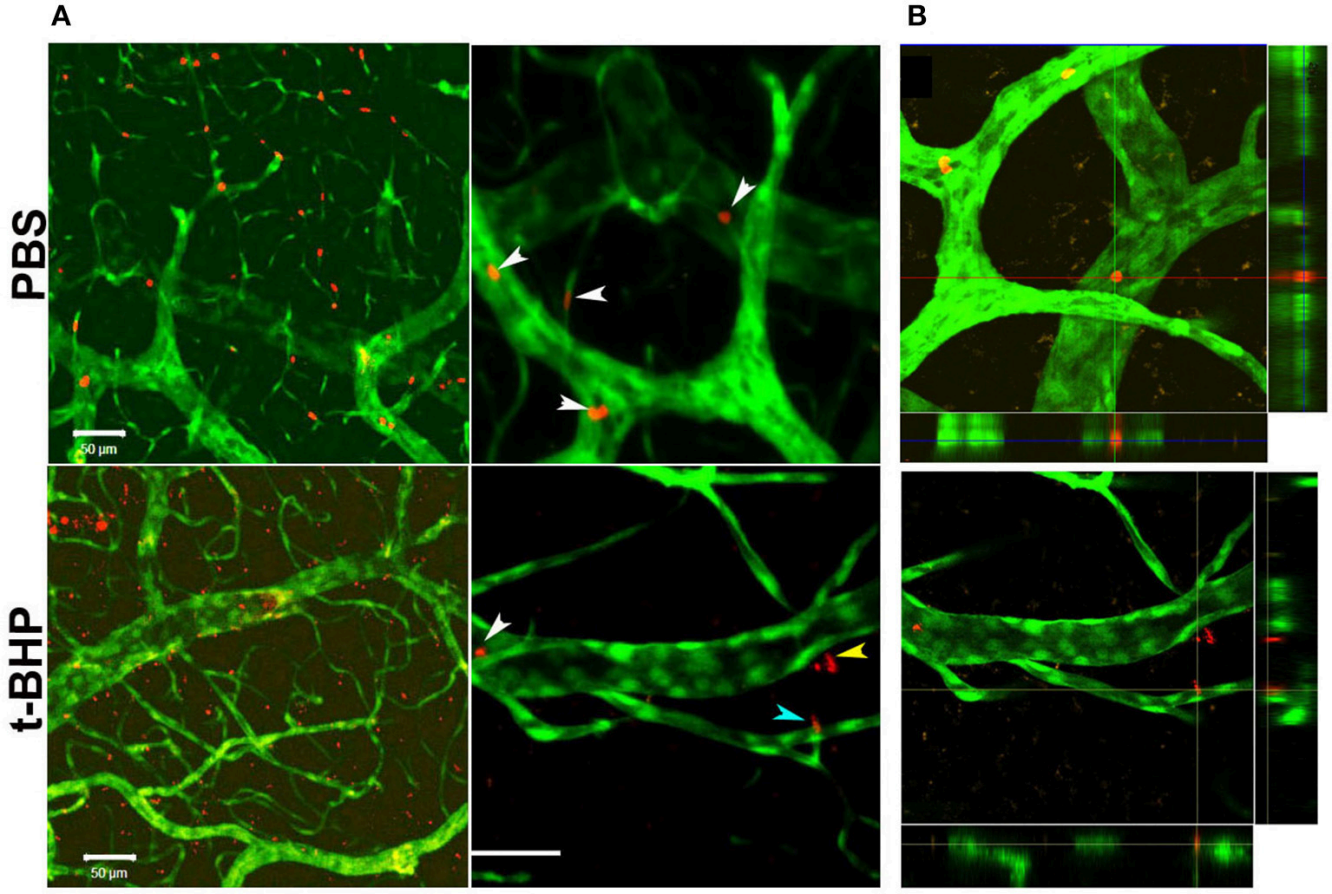
Postmortem confocal microcopy images on mouse brains showing GFP-positive brain endothelium (green) and PKH-26-labeled RBC (red). (**A**, Top): PBS-RBC remain in the blood vessels (white arrows heads). (**A**, Bottom): t-BHP-treated RBC are inside (white arrow head) and outside the blood vessels (yellow arrow head), and also partially extravasated into the brain (blue arrow head). Orthogonal view showing PBS-treated RBC within the blood vessel (**B**, Top) and t-BHP-treated RBC partially and completely outside the blood vessel (**B**, Bottom). Scale bar = 50 μm.

*In vivo* studies in Tie2-GFP mice showed that PKH-26 labeled PBS-treated RBC were confined to the cerebral vasculature. However, PKH-26 labeled t-BHP-treated RBC were found inside, outside, partially outside, and partially attached to the cerebral vasculature (Figure 5).

## Discussion

In this study we demonstrate that RBC exposed to the oxidative stressor t-BHP undergo significant erythrophagocytosis (adhesion and engulfment) by the brain endothelial cells in an *in vitro* cell culture system. Further, this enhanced erythrophagocytosis was associated with an increased passage of RBC degradation product hemoglobin across the brain endothelial cells. No change in brain endothelium monolayer integrity was observed with passage of hemoglobin across the endothelial monolayer in the current study. *In vivo* studies using Tie2-GFP mice further verified the passage of t-BHP-treated RBC across the brain endothelium.

Under normal physiological conditions, the adhesion of RBC to the vascular endothelium is negligible. However, altered RBC-vascular endothelial adhesion has been observed in various pathological conditions including sickle cell disease (SCD), diabetes mellitus, malaria, and retinal vein occlusion (Wautier and Wautier, 2013). Pathological adherence of RBC to the vascular endothelium is associated with vascular complications including vaso-occlusive events observed in SCD (Wautier et al., 1985). Further, cytoadherence of plasmodium falciparum-infected RBC to the brain microvascular endothelium has been demonstrated in cerebral malaria (Jambou et al., 2010; El-Assaad et al., 2013; Howland et al., 2015). Besides abnormal adhesion of RBC to the vascular endothelium, recent studies report endothelial erythrophagocytosis, a phenomenon by which aged and/or injured RBC adhere to and are engulfed by endothelial cells of peripheral origin including HUVECs and hepatic sinusoidal endothelial cells (Fens et al., 2010, 2012; Lee et al., 2011).

Among the first mechanisms shown to be involved in the phagocytic removal of aged, senescent, or injured RBC from the blood circulation (erythrophagocytosis) is membrane scrambling leading to the exposure of membrane PS to the outer leaflet of the RBC membrane (Bratosin et al., 1998). PS exposure serves as an “eat me” signal that triggers erythrophagocytosis by phagocytes, thereby facilitating erythrocyte clearance from the circulation (Schroit et al., 1985). Some of the seminal studies of RBC erythrophagocytosis showed that RBC with abnormal distribution of PS in the outer cell wall were phagocytized by macrophages more readily than normal or healthy RBC (Bratosin et al., 1998). PS-exposing cells are cleared from the circulation through recognition by receptors, including scavenger receptors, αvβ3 integrins, MerTK, TIM-1 and 4, BAI1, and Stabilin1 and 2, and subsequent attachment, cytoskeleton rearrangement and phagolysosomal processing (Ravichandran and Lorenz, 2007). The role of PS in endothelium-mediated erythrophagocytosis was also recently demonstrated (Lee et al., 2011; Fens et al., 2012). In these studies, PS-mediated endothelial erythrophagocytosis was mediated by the αvβ3 integrins (Fens et al., 2012) or stabilin 1 and 2 (Lee et al., 2011). Apart from endothelial erythrophagocytosis, PS exposure also plays a pivotal role in pathologic RBC adherence to vascular endothelium (Setty et al., 2002; Wautier et al., 2011).

In the current proof-of-concept study, PS exposure to mimic aged and/or injured RBC *in vivo* was triggered by treating RBC with t-BHP (Figure 1B) which, although an exogenous agent, is known to result in exposure of PS to the outer-leaflet of RBC (Fens et al., 2010), and is widely used as an external inducer of oxidative stress (Zou et al., 2001). Notably, oxidation of RBC plays an important role in erythrophagocytic clearance of aged and/or injured RBC (Kiefer and Snyder, 2000). As previously demonstrated by others, we found that a large percent of the t-BHP-treated RBC exposed PS (Fens et al., 2012). Consistent with studies using peripheral endothelial cells (Fens et al., 2012), in the current study we found that murine RBC treated with varying concentrations of t-BHP resulted in robust adhesion followed by engulfment of RBC by the murine brain endothelial cells (bEND3) (Figures 2, 3), a well-characterized immortalized brain microvascular cell line known to retain many morphological and biochemical properties similar to *in vivo* conditions (Omidi et al., 2003). Adhesion and engulfment of t-BHP-RBC was confirmed using multiple approaches (H&E, DAF assay, confocal analysis and live imaging). Erythrophagocytosis of t-BHP-RBC was not associated with loss or degradation of brain endothelial cells in the current study and is in agreement with a recent study using HUVEC cells, in which erythrophagocytosis of oxidative stress-exposed RBC did not result in immediate signs of degradation and only a small fraction of HUVEC cells showed signs of apoptosis after 24 h incubation (Fens et al., 2012).

Treatment of RBC with other pathological stimuli such as LPS or neuraminidase did not result in brain endothelial erythrophagocytosis or PS exposure (data not shown) in the current study. Further, activation of the brain endothelial cells with LPS did not augment erythrophagocytosis of t-BHP-treated RBC (Figure 1). These results are in contrast to previous studies in which treatment of RBC with LPS or neuraminidase resulted in significant adhesion to vascular endothelium (Tissot Van Patot et al., 1996; Eichelbrönner et al., 2000; Yang et al., 2014), and activation of endothelial cells with LPS further augmented such RBC-endothelial cell interactions *in vitro* (Tissot Van Patot et al., 1996; Eichelbrönner et al., 2000; Setty and Betal, 2008). The use of brain microvascular endothelial cells in the current study, as opposed to peripheral endothelial cells used in all the previous studies, may explain this difference in outcome.

After demonstrating robust erythrophagocytosis of RBC exposed to oxidative stress by the brain microvascular endothelial cells *in vitro*, the main question we wanted to address was whether brain endothelial erythrophagocytosis was associated with the passage of RBC or hemoglobin (RBC degradation product) across the brain endothelium. In the current study, we show significant passage of hemoglobin across the brain endothelial monolayer incubated with t-BHP-treated RBC (Figure 4). We further confirmed that the passage of hemoglobin was not associated with a change in the brain endothelial monolayer integrity since the TEER values remained unchanged for the duration of the experiment.

To evaluate the possibility of migration of free apical hemoglobin from t-BHP-treated RBC across the monolayer into the basolateral chamber, we determined the free hemoglobin released by t-BHP-RBC compared with PBS-RBC over 24 h in a separate series of experiments using bEND3 monolayers grown in standard cell culture plates (not transwells). We found that t-BHP-treated RBC did not release more free hemoglobin into the media compared with the PBS-RBC, and there was significantly less free hemoglobin in the media of brain endothelial cells incubated with t-BHP-RBC compared with PBS-RBC (Figure 4) following 18 h and 24 h incubation. This suggests that the hemoglobin in the basolateral chamber does not represent the migration of free hemoglobin released in the apical chamber. The decrease in free hemoglobin with t-BHP-RBC at 18 h and 24 h is consistent with increased RBC erythrophagocytosis of t-BHP-RBC by brain endothelial cells, and thus lower free RBC available to release hemoglobin at these time points.

A limitation of our *in vitro* findings is the use of an immortalized brain endothelial cell line. Note, however, that passage of RBC degradation product (hemoglobin) across the brain endothelial monolayer were corroborated by our *in vivo* findings of extravasation of fluorescently-labeled t-BHP-treated RBC across the brain endothelium of Tie2-GFP mice using confocal microscopy (Figure 5). We reported a similar finding of extravasation of fluorescently-labeled RBC across the brain endothelium of Tie2-GFP mice in an inflammation-induced mouse model of CMB (Sumbria et al., 2016). In the current study, the blood vessel adjacent to the extravasated RBC appeared intact with no visible rupture (Figure 5).

Erythrophagocytosis mediated by macrophages involves RBC engulfment, endosomal processing and RBC degradation with subsequent release of hemoglobin and heme. It should be noted that not all engulfed RBC are subjected to phagosomal degradation and that the fate of the engulfed RBC depends on the molecular machinery involved in erythrophagocytosis (Santarino et al., 2017). The intracellular mechanisms involved in the cellular trafficking of engulfed RBC and subsequent RBC processing by endothelial cells are understudied.

In the current study, we report the passage of RBC degradation product hemoglobin *in vitro* and the passage of fluorescently-labeled RBC across the brain endothelium *in vivo*. The possibility of passage of RBC fragments and not intact RBC cannot be ruled-out from the current data. We are unaware of prior reports of extravasation of non-parasitized RBC across the brain endothelium. Our findings are similar to the work done by Grutzendler and co-workers who reported engulfment and subsequent extravasation of emboli into the brain (termed as angiophagy; Grutzendler et al., 2014). Following passage across the brain endothelium, emboli were engulfed and degraded by pericytes and microglial cells. Future *in vitro* studies using co-cultures with pericytes and microglial cells will help elucidate the intracellular trafficking and the fate of the RBC involved in brain endothelial erythrophagocytosis.

**Figure 6:**
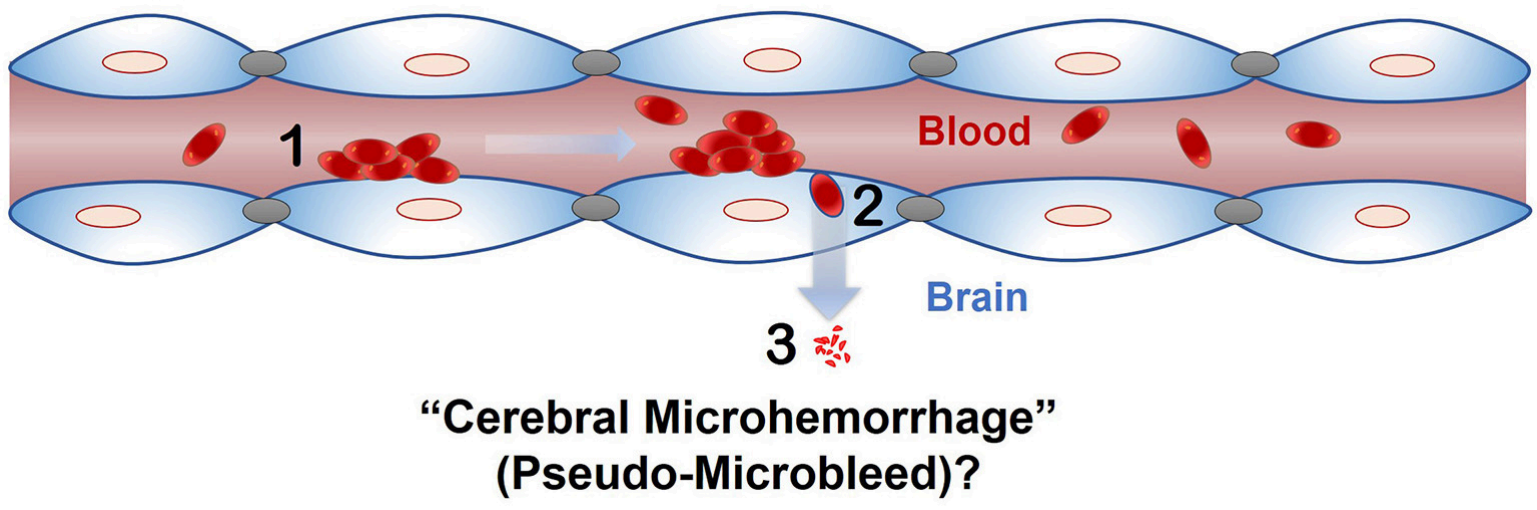
Proposed role of endothelial erythrophagocytosis in cerebral microhemorrhage (pseudo-microbleeds) development. Aged and/or injured RBC adhere to (1) and are engulfed by (2) the brain endothelium. Iron rich RBC or degradation products (e.g., hemoglobin) are translocated across the brain endothelium (3), where they may produce signatures of CMB.

The phenomenon of passage of intact RBC or RBC degradation products across an intact endothelial monolayer following endothelial erythrophagocytosis is a novel finding, and brain endothelial erythrophagocytosis is particularly relevant from the standpoint of non-hemorrhagic mechanisms involved in CMB development (Figure 6) (Fisher, 2014). This is because CMB are focal deposits of blood degradation products, predominately hemosiderin, which is derived from hemoglobin following hemolysis (Janaway et al., 2014). MRI detection of CMB relies on the paramagnetic properties of hemosiderin and any passage of hemoglobin across the brain endothelium into the perivascular space can thus produce signatures of CMB. Further investigation is required to determine whether brain endothelial erythrophagocytosis and subsequent passage of RBC or RBC degradation products across the brain endothelium occur under physiologically relevant conditions that induce oxidative stress *in vivo* to produce CMB signatures. Notably, PS exposure on erythrocytes and oxidative stress has been reported under several risk factors for CMB, such as chronic kidney disease (Bonomini et al., 1999, 2002; Tucker et al., 2015) and Alzheimer's disease (Nicolay et al., 2007; Gella and Durany, 2009). Further, CMB in the absence of gadolinium extravasation (marker of blood-brain barrier disruption) have been reported in patients with cerebral malaria (Potchen et al., 2018), and recent work showed engulfment of parasitized RBC by human brain endothelial cells in an *in vitro* model of cerebral malaria (Jambou et al., 2010), raising the possibility of erythrophagocytosis-dependent CMB in this disorder.

In conclusion, we show that brain endothelial cells express an erythrophagocytic phenotype for RBC exposed to oxidative stress *in vitro*. Further, brain endothelial erythrophagocytosis is associated with passage of hemoglobin across the brain endothelial monolayer *in vitro*, and passage of RBC exposed to oxidative stress across the brain endothelium *in vivo*. These results may have significant implications for mechanisms of CMB in the absence of frank vascular disruption.

## Data availability statement

All datasets for this study are included in the manuscript and the Supplementary Video 1. The raw data supporting the conclusions of this manuscript will be made available by the authors, without undue reservation, to any qualified researcher.

## Author contributions

RC performed the experiments, collected and analyzed data, and contributed to manuscript writing. JC performed the experiments and collected data. TK performed confocal microscopy for *in vivo* studies. AZ helped with live imaging. MF helped conceive the study and edited the manuscript. RS conceived the study, designed, analyzed and coordinated the experiments, and drafted and edited the manuscript. All authors read and approved the final manuscript.

### Conflict of interest statement

MF has received support from Boehringer-Ingelheim and Otsuka Pharmaceutical Company (research grants). The remaining authors declare that the research was conducted in the absence of any commercial or financial relationships that could be construed as a potential conflict of interest.

## Supplementary material

Supplementary Video 1 Live cell imaging of brain endothelial erythrophagocytosis. The highlighted region shows attachment and subsequent engulfment of two RBC by brain endothelium.
